# Toxic heavy metals distribution in urban soils of Africa: a systematic review

**DOI:** 10.1007/s10661-026-15030-9

**Published:** 2026-02-05

**Authors:** Nontokozo Pertunia Mkhonza, Sandisiwe Zondo, Samukelisiwe Vilakazi

**Affiliations:** 1 Discipline of Agriculture, School of Agriculture and Science, University of KwaZulu-Natal, P.O. Bag X01, Pietermaritzburg Campus, Scottsville, 3209 South Africa; 2 Soil Fertility and Analytical Services Division, KwaZulu-Natal Department of Agriculture and Rural Development, Private Bag X9059, Pietermaritzburg, 3200 South Africa; 3 Department of Agriculture, Cape Peninsula University of Technology, Wellington, Western Cape, 7655 South Africa

**Keywords:** Heavy metals, Industrialisation, Soil pollution, Toxic elements, Urbanisation

## Abstract

Urban soils in Africa are increasingly contaminated by toxic heavy metals due to rapid urban expansion, industrialisation, traffic emissions, and inadequate waste management. Toxic heavy metals such as lead (Pb), mercury (Hg), cadmium (Cd), arsenic (As), chromium (Cr), and nickel (Ni) are of major concern because of their persistence, bioaccumulation potential, and ecological risks for the environment and humans. This systematic review synthesises evidence from 38 peer-reviewed studies published between 2010 and 2024 on the concentrations and sources of toxic heavy metals in urban soils of Africa. The results demonstrated an uneven research distribution on toxic heavy metals across different countries of Africa, with Nigeria and Ghana most represented, while large regions, including countries such as Egypt and Ethiopia, remain poorly studied. Industrial activities (27%) and traffic emissions (26%) accounted for more than 50% of the reported contamination sources, followed by domestic waste disposal (18%) and agricultural inputs (10%). Weighted mean concentrations of Pb, Cd, and Cr in many countries exceeded FAO permissible limits, indicating a significant threat to humans and the environment. Microwave digestion was the most commonly used extraction method, while X-ray fluorescence has gained increasing application. The findings demonstrate widespread contamination from rapid urbanisation and industrialisation but highlight limited research done on toxic heavy metals in urban areas of most African countries. Future research should focus on quantifying the metal concentration in African countries, where limited work has been done; the bioavailable fractions of toxic heavy metals and possible remediation strategies to improve soil quality in urban areas.

## Introduction

On a global scale, urban soils have become critical sinks for toxic heavy metals resulting in contamination (Li et al., 2018). The exponential increase in population (Imperato et al., 2003; Shi et al., 2023) exacerbates this problem due to industrialisation and rapid urbanisation. Urban soils often differ from those from non-urban areas in their chemical and physical properties, often characterised by degraded soil structure and elevated concentrations of toxic heavy metals (Manta et al., 2002). Heavy metals such as lead (Pb), cadmium (Cd), chromium (Cr), copper (Cu), zinc (Zn), nickel (Ni), arsenic (As), and mercury (Hg) are of concern due to their persistence, non-biodegradability, and potential for bioaccumulation in ecosystems and human food chains (Shi et al., 2023). A recent global analysis report demonstrated that approximately 14 to 17% of cropland is contaminated by one or more toxic metals at levels exceeding the permissible thresholds, with Cd particularly prevalent in parts of Africa, threatening both environmental and human health (Hou et al., 2025). In a global meta-analysis , the concentration of Cd in African urban soils ranged between 0.03 and 299 mg kg^−1^ with an average of 22.6 mg kg^−1^; which exceeded the permissible levels in soils (Adewumi & Ogundele, 2024). In an African context, studies across diverse urban environments have highlighted the severe effects of heavy metal contamination in both urban and agricultural areas.

In Nairobi, Kenya, peri-urban soils used for vegetable farming demonstrated elevated concentrations of several toxic heavy metals, particularly Cd, Pb, and Zn, which exceeded permissible levels, and ecological risk analyses indicated that Cd contributed to 46 to 66% of the total risk burden (Ahogle et al., 2023). In Nigeria, residential soils in Lagos and Ibadan cities demonstrated significantly higher concentrations of Pb (up to 419 mg kg^−1^) and Cd, when compared to the thresholds, particularly in vulnerable low-income settlements (Adeyi & Babalola, 2017). These elevated concentrations and associated risks highlight the importance of understanding the soil processes that govern heavy metal mobility and persistence. It is evident that toxic heavy metals are soluble in soils and could increase the likelihood of bioaccumulation within the food chain (Angon et al., 2024). To contextualise this, it is essential to note that the solubility, mobility and bioavailability of heavy metals is regulated by soil chemical properties.

The distribution, mobility, and toxicity of heavy metals in soils are strongly influenced by soil chemical properties such as soil pH, which plays a central role in controlling metal solubility, with acidic conditions generally increasing metal mobility and availability for plant uptake (Adriano, 2001). Moreover, clay minerals, particularly smectites and illites, enhance heavy metal adsorption via cation exchange and surface complexation, consequently reducing mobility (Alloway, 2012). Similarly, soil organic matter can immobilise heavy metals by forming stable organo-metallic complexes. However, under anaerobic or acidic conditions, organic ligands may enhance metal solubility (Tack & Verloo, 1995; Kabata-Pendias, 2011). Redox potential is equally critical, as fluctuating conditions in urban wetlands and drainage areas can trigger the mobilisation of metals such as Fe, Mn, and associated trace elements (Shahid et al., 2019). These interactions between the soils and toxic heavy metals highlight the complexity of heavy metal dynamics in urban soils and highlight the importance of site-specific assessments. Heavy metal contamination in urban soils poses a complex risk to both the ecosystem and humans (Ayaz et al., 2023; Monib et al., 2024). High concentrations of heavy metals in soils can significantly disrupt microbial populations, reduce nutrient cycling, and overall soil quality (Naz et al., 2022; Wang et al., 2022).

High concentrations of Pb, Cd and Cr have been linked to reduced biodiversity and altered biogeochemical processes in urban soils (Ayaz et al., 2023; Musah, 2024). These changes can cascade through the food web, affecting soil fauna and plant health and increasing the risk of bioaccumulation in crops grown in contaminated soils. Humans are exposed to contaminants through ingestion of contaminated soil, consumption of crops grown on contaminated soil, inhalation of contaminated dust, or leaching into groundwater (Economou-Eliopoulos & Megremi, 2021; Valskys et al., 2022). The health consequences of toxic heavy metals are well documented in literature. For instance, Pb is neurotoxic, particularly in children; Cd can damage renal systems; and Cr and As are recognised carcinogens (Sharma & Kumar, 2025). In many African cities, informal settlements and urban agriculture coexist alongside contamination hotspots, increasing the risk of exposure for vulnerable populations (Zerbo et al., 2020). Despite the evidence demonstrated in a global meta-analysis study synthesising heavy metal distribution in urban soils, including certain African regions (Adewumi & Ogundele, 2024), Africa is significantly under-represented in these evaluations. Existing research is fragmented, dominated by studies from a few countries, and characterised by inconsistent sampling and analytical methods. Current research on heavy metals in urban soils in Africa is primarily focused on individual cities, and frequently overshadowed by data from Asia, Europe, and North America (Adewumi & Ogundele, 2024). Moreover, no comprehensive analysis has specifically investigated the distribution of heavy metals in urban soils across Africa, despite the region's rapid urbanisation, inadequate environmental monitoring systems, and different soil chemical characteristics that affect metal mobility. This fragmentation hinders thorough risk evaluation and the establishment of appropriate measures. Therefore, the objective of this study was to synthesise the available literature on total heavy metal sources, contamination and distribution in urban African soils.

## Materials and methods

### Data sources and search strategy

**Fig. 1:**
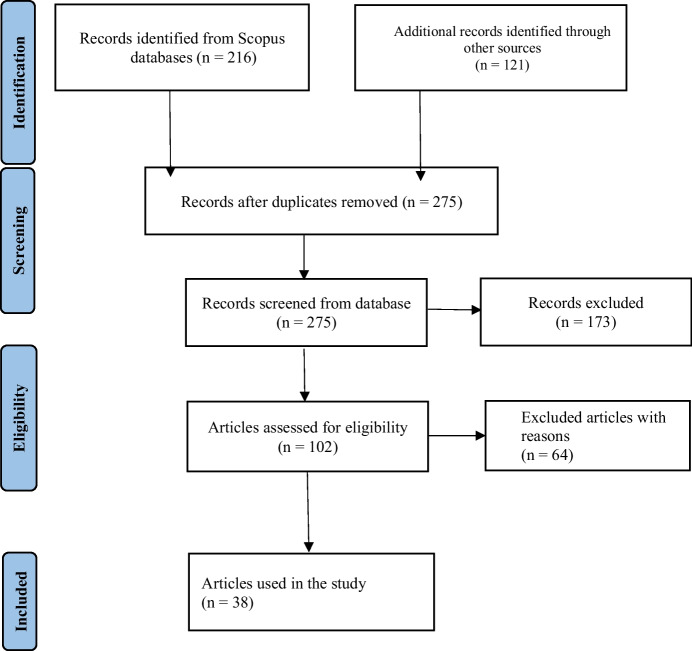
PRISMA flow diagram adapted for article selection protocol for Systematic Reviews and Meta-analysis Protocols (PRISMA-P) 2015 statement

This review focused only on urban soils in Africa with 38 studies in total published over the last 14 years after exclusion of irrelevant studies (Table 1). Different search terms were used to include and exclude studies (Table 2). The exclusion and inclusion criteria used were based on PRISMA flow diagram for systematic reviews and meta-analysis (Fig. 1). Secondary data were extracted from relevant peer-reviewed articles using different search engines: Scopus (accessed date 21 March 2025), Web of Science (22 March 2025), Science Direct (22 March 2025), Google scholar (23 March 2025). Only articles published in English were considered.

**Table 1:** Boolean operators used for each database to identify peer-reviewed articles examining heavy metals distribution in urban soils in Africa

Database (s)	Primary term (s)	Search term (s)
Scopus, Web of Science, Science Direct, Science.gov and Google Scholar	Urbanisation AND Industrialisation	"urbanisation" OR "urban expansion" OR "urban growth" OR " industrialisation" OR "industrial development" AND
	Soil pollution AND Contamination	"pollution" OR "soil contamination" OR "soil quality" OR "soil degradation" OR "urban soils" AND
	Heavy metals	" metals" OR "trace metal" OR "trace elements" OR "toxic metals" OR "metal contamination" OR "metal pollution" OR "lead" OR "cadmium" OR "arsenic" OR "mercury" OR "chromium" OR "nickel" AND
	Urban areas	"urban areas" OR "cities" OR "metropolitan regions" OR "urban soils"

**Table 2:** Groupings of different source of heavy metals

Source (s)	Group (s)
Industrial activities; Industrialisation; Manufacturing; Processing industries; mining; metal works; electroplating industries; waste incineration	Industrial Activities
Vehicle emissions; vehicular emissions; traffic emissions; vehicles; auto mechanic activities; combustion and corrosion; roadside contamination; road traffic and power generating sites	Traffic and Transport
Agricultural activities; fertiliser use; pesticide and herbicide use; irrigation water; livestock waste	Agriculture and Agrochemicals
Domestic waste; household and commercial waste; waste disposal; solid waste dumps; municipal waste; open dumping sites	Domestic and Municipal Waste
Informal recycling of electronic waste; battery waste; burning of e-waste, used electronic equipment	E-waste and Informal Recycling
Urbanisation; construction activities; land use change; building materials; historical pollution from development	Urbanisation and Construction
Geological sources; natural background weathering of rocks	Natural Sources

### Research question (s)

To ensure relevant studies were used, the following questions were used: (i) What is the distribution of total heavy metals in urban soils of Africa? (ii) What are the major sources of heavy metals in urban soils of Africa?

### Exclusion and inclusion criteria

The following criteria were used to exclude articles which were not relevant to this study: sediment and dust samples; results from outside urban areas; bioavailable heavy metals fraction; results obtained through geographic information system (GIS) and modelling; agriculture (unless its urban agriculture reporting total heavy metals concentration); mine sites; conference papers; books and reviews. The GIS and modelling data were excluded because they rely on secondary datasets and assumptions may introduce uncertainty or bias. The articles were restricted to publication dates between 2010 and 2024. This time restriction was used to ensure retrieval of most recent data on the distribution of heavy metals in urban soils of Africa. For the inclusion criteria peer-reviewed articles which reported work on total heavy metals in urban areas including schools, roadside, parks and urban gardens were used in this review. The heavy metals reported in this work were Pb, Cd, As, Hg, Cr and Ni. In cases where an article reports other heavy metals, the other metals were excluded.

### Data classification, summary, and reporting

The data from the studies, that were included, were systematically extracted into a structured database consisting of source information (author, year, country and region), characteristics of the study (analytical technique and extraction methods), contamination sources, and reported total concentrations of the target heavy metals (Pb, Cd, Cr, As, Hg, and Ni). Excel was used to determine the frequency of studies by country, publication patterns, and the proportional contribution of contamination sources using the collected information in the database. Frequency counts and percentages were used to determine the distribution of extraction methods and contamination sources among the reviewed studies. The countries were categorised into North, West, East, Central, and Southern Africa to enable regional comparisons (Table 3). Where studies report heavy metal concentration ranges, both minimum and maximum values were recorded, while mean or median data were used where reported in the results.

**Table 3:** Summary of studies on heavy metals distribution in urban soils of Africa

Authors	Source of Heavy Metal	Extraction and analytical method	Statistical tool	Total heavy metals (mg kg^−1^)	Implications	Country	Country Grouping
Naylo et al., 2019	Vehicle Emissions; Combustion and Corrosion; Building Materials and Technogenic Fraction	Microwave digester/ICP-OES	SPSS 21.0	As = 8.64; Cd = 0.229; Cr = 27.6; Ni = 16.4; Pb = 32.6. n = 27	The findings suggest a need for continuous monitoring to prevent excess toxic elements in urban soils, ensuring public safety	Morocco	North Africa
Konwuruk et al., 2021	Agricultural Activities; Industrial and Artisanal Activities; Vehicular Emissions; Municipal Waste and Sewage and burning of municipal waste	X-Ray Fluorescence/ICP-MS	-	As = 10.11; Cd = 12.91; Cr = 77.97; Pb = 18.60; Ni = 29.33. n = 73	The study implies that while current health risks are not significant, continuous monitoring is essential to prevent future bioaccumulation and associated health risks	Ghana	West Africa
Nyandika et al. (2020)	Domestic and industrial wastes; Municipal and industrial drainage; sewage sludge and irrigation with wastewater	X-Ray Fluorescence Spectrometer	R 3.3.3	Cr = 58.1; Ni = 30.6 n = 15	Monitoring trace elements in urban farming soils is crucial to prevent contamination and ensure ecosystem health, as these elements can enter the human food chain through plants	Kenya	East Africa
Benhaddya et al., 2016	Traffic Emissions and Industrial Activities	Microwave digester/Atomic absorption spectrometer	SPSS 16.0	Ni = 38.5; Pb = 180. n = 38	The findings suggest a need for monitoring and managing heavy metal pollution in urban environments to protect human health, particularly for vulnerable groups like toddlers	Algeria	North Africa
Darko et al. 2017	Industrial Activities; Auto Mechanic and Metal Scrap Recycling; Electroplating and Fuel Burning	X-Ray Fluorescence Spectrometer and Microwave digester/ICP-MS	SPSS 20.0	As = 4.59; Pb = 53.5; Ni = 43.2; Cr = 125; Hg = 0.28. n = 107	The findings imply that there is a need for monitoring and managing soil contamination in urban areas to mitigate health risks to urban dwellers and protect the ecosystem. The study also highlights the potential of using FP-XRF for fast measurements of heavy metals in soils, which could be beneficial for ongoing monitoring efforts	Ghana	West Africa
Kanda et al., 2018	Activities like welding, automobile maintenance, and waste dumping contributed significantly to contamination	Microwave digester/Atomic absorption spectrometer	SPSS 21	Cd = 1.42; Cr = 82.0; Pb = 99.4. n = 20	Paving surfaces may reduce trace element dispersal and human exposure. Legislation and enforcement in the informal industrial sector are needed to safeguard health and environmental quality	Zimbabwe	Southern Africa
Iwegbue and Martincigh (2018)	Industrial emissions; Vehicular abrasion and exhaust; Traffic-related wear; Fossil fuel combustion; Industrial and municipal waste; Residential activities	Microwave digester/Atomic absorption spectrometer	-	Cd = 0.63; Pb = 45.4; Cr = 22.4; Ni = 7.77. n = 82	The study highlights the need for urban planning and management to incorporate health risk assessments to ensure a sustainable environment	Nigeria	West Africa
Boudia et al., 2019	Industrial Activities; Traffic: Vehicular emissions	Microwave digester/ICP-AES	SPSS 24.0.0	As = 10.9; Cr = 55.0; Ni = 27.5; Pb = 52.2. n = 67 excluding agricultural zones	The findings highlight the need for local authorities to conduct more extensive studies and consider remediation efforts to mitigate health risks from soil contamination	Algeria	North Africa
Sellami et al., 2022	Vehicle emissions; Industrial activities; Waste disposal; Animal feces and Fertilizers	Microwave digester/Atomic absorption spectrometer	Golden software Surfer 16	Cd = 0.02; Cr = 43.4; Pb = 331. n = 36	The accumulation of heavy metals from anthropogenic sources poses environmental and health risks	Algeria	North Africa
Darko et al. 2017	Disposal of electrical and electronic equipment; Use of paints and pesticides; Incineration of domestic waste; Industrial activities; Fossil fuel combustion; motor oil spills	Microwave digester/ICP-MS	Minitab 17	As = 9.50; Cd = 0.64; Cr = 124; Hg = 0.05; Ni = 10.6 and Pb = 97.0. n = 107	Incorporating bio accessibility data can provide a more accurate estimation of health risks associated with soil ingestion, highlighting the importance of considering bioavailability in risk assessments	Ghana	West Africa
Famuyiwa et al., 2018	Industrial Activities; Vehicular Emissions; Waste Disposal; Historical Pollution and Urbanisation and Construction	Microwave digester/ICP-MS	-	Cr 82.2; Ni = 32.5; Pb = 173. n = 126	The study emphasizes the need for stricter environmental regulations, waste management strategies, and emission controls to mitigate pollution	Nigeria	West Africa
Amponsah et al., 2022	Primary source was informal recycling of electronic and electrical waste (E-waste)	X-Ray Fluorescence Analyzer and Microwave digester/ICP-MS	R tool	As = 45.5; Cd = 5.1; Cr = 119; Hg = 0.566; Ni = 52.9 and Pb = 780. n = 31	High bio accessibility of metals like Pb, Cd, and Ni suggests significant health risks for local populations. The findings highlight the need for regulatory measures to manage E-waste recycling practices	Ghana	West Africa
Lala et al., 2022	Vehicles; Power generating sets; Petrol stations; Machine workshops	Microwave digester/Atomic absorption spectrometer	Microsoft Excel and XLSTAT	Cr = 660 and Pb = 360. n = 24	The contamination of university soils by trace metals poses significant risks and hazards to the immediate ecosystem if not properly managed. Periodic assessment of the sources and associated ecological risks of the heavy metals is highly recommended to enable decision-makers to effectively manage the environment and preserve public and ecosystem health	Nigeria	West Africa
Addis et al. 2025	Geological Formations; Industrial activities; Sewage, Solid waste dumping; Automobile exhaust; Auto-mechanical works	Microwave digester/ICP-MS	Minitab 17	Cr = 77.3; Cd = 0.24; Ni = 62.7; Pb = 24.3. n = 36	The findings suggest that the allowable concentrations of trace elements in agricultural soils may need to be more stringent to prevent health risks. The study highlights the need for restrictions on irrigation water quality and the consumption of vegetables cultivated in urban soils with high levels of toxic metals. It underscores the public health risks associated with urban vegetable farming in areas with polluted water sources, emphasizing the need for regulatory measures and public awareness	Ethiopia	East Africa
Gyimah et al. 2022	Automobile mechanic shops	Microwave digester/Atomic absorption spectrometer	SPSS 22.0	Approximate: Cr = 7.33; Pb = 44.8; Ni = 69.0; Cd = 0.48. n = 20	Highlights the need for monitoring and remediation efforts to protect human health and the environment from heavy metal contamination	Ghana	West Africa
Kolawole et al., 2023	Industrial activities; Municipal solid waste landfill	Microwave digester	SPSS	As = 1.20, Cr = 86.3, Pb = 130, Ni = 22.3, Cd = 1.78. n = 64	The findings highlight the need for monitoring and managing soil contamination in urban areas to mitigate ecological and health risks	Nigeria	West Africa
Bellarbi et al., 2015	Urban activities; Traffic emissions; Industrial Sources; Agricultural Sources; The use of wastewater for irrigation	Microwave digester/ICP-AES/ICP-MS	-	Cr = 75; Ni = 41.3; Pb = 64.8. n = 3	The findings highlight the need for better pollution control and wastewater management in Fez to prevent environmental risks and ensure safe agricultural practices	Morocco	North Africa
Adedeji et al., 2019	Anthropogenic sources; including emissions from artisan workshops, road traffic, and incineration at dumpsites. The use of leaded paints in artisan workshops	Microwave digester	SPSS 17.0 and Excel 2007	Pb = 82.1; Cd = 3.07; Ni = 7.03; Cr = 1.80. n = 72	The findings suggest a need for proper control and monitoring of urban activities that release heavy metals into the soil to safeguard human health	Nigeria	West Africa
Olorundare et al., 2011	Mechanic workshops	Microwave digester/Atomic absorption spectrometer	-	Cr: 2.08. n = 32	The findings highlight the need for environmental regulations to manage waste from artisan activities to protect public health and the environment	Nigeria	West Africa
Okosa et al., 2023	Industrial and urban waste sites	Hot plate	-	Cr = 0.64; Pb = 0.22; Ni = 1.62 and Cd = 0.61. n = 40	The findings highlight the need for immediate evacuation and remediation to prevent further degradation and potential groundwater contamination	Nigeria	West Africa
Akinsete et al., 2021	Urban activities	Microwave digester	-	Pb = 84; Ni = 6	There is an urgent need for actions to safeguard soil environments, especially considering climate change impacts	Nigeria	West Africa
Luilo et al. 2014	Traffic emissions	Microwave digester/Atomic absorption spectrometer	-	As = 0.23. n = 30	The findings highlight the need for monitoring and managing urban soil contamination to mitigate public health risks associated with arsenic exposure	Tanzania	East Africa
Beroigui et al., 2020	Airborne Deposits; Industrial activities. This includes emissions from road traffic	Microwave digester/ICP-OES	SPSS 10	As = 6.39; Cr = 19.0; Ni = 15.6; Pb = 56.4; Cd = 0.52. n = 18	The findings highlight the need for monitoring and managing soil quality in urban areas to protect public health, especially for vulnerable populations like children	Morocco	West Africa
Maas et al., 2010	Industrial emissions; Traffic; Agricultural activities	Microwave digester/Atomic absorption spectrometer	R 2.5.1, gstat 09.−39 and pgirness 1.3.3	Cd = 0.44; Cr = 30.9; Pb = 53.1. n = 101	The study highlights the need for human and environmental risk assessments in areas with high metal concentrations, such as those with elevated Cd and Pb levels, to determine the necessity of remediation efforts	Algeria	North Africa
Ahogle et al., 2023	Industrial waste; Urban emissions; Mining operations; Sewage sludge and Wastewater	Microwave digester/ICP-MS	R 4.1.2	As = 3.61; Cd = 0.22; Cr = 13.6; Pb = 19.2; Ni = 11.8; Hg = 19.3. n = 60	The results indicate a need for regular monitoring and management of contaminated soils to ensure clean and safe food production in urban soils	Kenya	East Africa
Shezi et al., 2022	Industrial operation; Exhaust vehicular emissions	X-Ray Fluorescence	Stata IC 14	As = 16; Pb = 30. n = 34	The results showed variability in the source of heavy metals, indicating a need for monitoring this contamination in soils over time	South Africa	Southern Africa
Konadu et al., 2023	Automative repairs associated with auto mechanics workshops	X-Ray Fluorescence	R 4.2.2 and Minitab 16.2.1	As = 54.3; Cd = 54.6; Cr = 65.7; Ni = 564; Pb = 88.4. n = 131	The findings indicate that the high concentrations of metals such as Co, Ni, and Pb pose potential health risks to the exposed populations in the Suame and Asafo areas. This underscores the importance of monitoring and regulating industrial activities, particularly in areas with a high concentration of auto mechanic workshops, to mitigate health risks associated with toxic metal exposure	Ghana	West Africa
Hareinda et al. (2023)	Effluent discharge, wastewater infiltration, landfills, composts	Microwave digester/Atomic absorption spectrometer	Statgraphics Centurion XVI	Cd = 2.98; Pb = 146; Ni = 35.2. n = 9	The contamination levels indicate a substantial risk of ecological harm and possible human exposure, particularly if these soils are used for agricultural activities. The authors stress that the elevated levels of these metals, especially in combination with the acidic soil pH which enhances metal mobility and uptake by plants, render these soils unsuitable for food production. Continued use of these lands without remediation could result in the entry of toxic metals into the food chain, posing long-term public health risks	Cameroon	Central Africa
Silva et al., 2020	Industrialisation	Microwave digester/ICP-OES/ICP-MS	ANDAD 7.11	As = 0.0045; Cd = 0.00056; Cr = 0.023; Hg = 0.045; Ni = 0.011; Pb = 0.033. n = 24	Lead poses a potential health risk to children exposed to unpaved urban areas. Although cadmium’s non-cancer risk was low, its presence is concerning. Urban expansion into floodplains and inadequate waste management are key contributors	Angola	Southern Africa
Mama et al., 2022	Vehicular Emissions and Traffic-Related Activities; Auto Mechanic Workshops and Vulcanizing Shops; Roadside Open Dumpsites and Waste Burning; Industrial and Urban runoff	Microwave digester/Atomic absorption spectrometer and UV–Visible spectrophotometer	-	Pb = 0.58; Cd = 0.00259; As = 3.33; Ni = 0.547; Cr = 0.413. n = 21	The study highlighted the need for regular monitoring of the heavy metals’ concentrations in roadside soils, due to urban areas and high vehicular activity and industrial operations which causes accumulation of heavy metals which can move through dust to the soils	Nigeria	West Africa
Omonona & Okogbue, 2024	Agriculture was the main source in the suburban areas (fertilizers and pesticides); Urban and industrial pollution; Wastewater runoff	Microwave digester/Atomic absorption spectrometer	Statgraphics Centurion XVI.I	Pb = 52.5; Ni = 5.5; As = 0.8; Cr = 20; Hg = 0.18. n = 25	The study highlighted the potential contamination of ground water through leaching of the trace elements especially in soils which are highly permeable, threatening drinking water quality	Nigeria	West Africa
Githaiga et al., 2021	Intensive traffic; Industrialisation; Urbanisation; Solid waste	Microwave digester/X-ray fluorescence spectrometer and ICP-MS	-	Pb = 10.8; Cd = 0.67; Cr = 27; As = 26; Ni = 16.3. n = 7 (urban lands only)	The study highlights the need for integrated managed of agriculture and industrial waste production to mitigate pollution	Kenya	East Africa
Konadu et al., 2023	Automotive activities; anthropogenic inputs; Industrial and traffic emissions	XRF Fluorescence	R tool 4.2.2	As = 54.3; Cd = 54.6; Cr 65.7; Ni 564. n = 131	The study highlighted the potential toxic elements in soils which exceeded the permissible levels and highlighted the urgent need for remediation strategies such as stricter regulations, and proper waste management in auto mechanistic workshop	Ghana	West Africa
Adewumi, 2022	Urbanisation; Industrialisation; Vehicular emission; Waste disposal; construction and quarrying	Microwave digester/Atomic absorption spectrometer	SPSS 23.0	As = 1.67; Cd = 1.87; Cr = 27.7; Ni = 10.1; Pb = 34.3. n = 16	The levels of Cd in the soils are above the permissible levels indicating potential environmental contamination	Nigeria	West Africa
Sako et al., 2023	Mining; Waste disposal	Microwave digester/X-ray fluorescence spectrometer and atomic absorption spectroscopy	IBM SPSS 20	Cr = 65.5; As = 11.6; Ni = 21.1; Pb = 3012. n = 30	The study highlights the environmental and health hazards posed by improper waste management in gold analysis laboratories. The findings call for stricter enforcement of environmental policies and public health interventions in similar settings	Burkina Faso	West Africa
Essel, 2017	Agrochemicals (pesticides, herbicides, fertilizers); Industrial and Mining; Overgrazing, deforestation, and bush fires; Urbanisation and Waste disposal	Microwave digester/X-ray fluorescence spectrometer	-	Cr = 385; Pb = 37; Cd = 3.2. n = 20	Improved farming practices, regulating artisanal mining procedures, preservation of natural resources, among others, may help minimize degradation of the environment	Ghana	West Africa
Egbueri et al., 2022	industrial processes (e.g., chemical manufacturing, e-waste, rubber/plastic waste); automobile workshops and historical use in batteries/fuels; geology of the area does not naturally enrich these metals	Microwave digester/Atomic absorption spectrometer	-	Pb = 0.005; Ni = 0.002; Cr = 0.029; Cd = 0.220. n = 21	While current pollution levels are mostly low, Cd and other heavy metals (Ni and Cr) require urgent attention to prevent long-term ecological and health impacts. Effective waste management, regulatory enforcement, and community engagement are key to sustainable mitigation	Nigeria	West Africa
Okorie et al. 2024	Industrial Activities; Agricultural Practices; Improper Waste Disposal; Urbanisation and Construction; Vehicular Emissions; Household and Commercial Waste	Microwave digester/Atomic absorption spectrometer	SPSS 20.0 and Microsoft Excel 2016	Pb = 0.34; As = 0.393; Cr = 1.19. n = 6	The study highlights the importance of continuous monitoring and environmental regulations to ensure sustainability	Nigeria	West Africa

### Data standardisation and presentation

This review used total concentration of heavy metals in the different studies. The mean of heavy metals concentration was standardised to mg kg^−1^ and, where overall mean was not reported, the different observations were averaged. This was done to allow accurate comparison and discussion of the results. Visual summaries of the results were created in Microsoft Excel using bar charts, trend lines, and pie charts to illustrate (i) the publication count over the years, (ii) the geographic distribution of studies across Africa, (iii) the extraction method and analytical technique used across studies, and (iv) the different anthropogenic sources of heavy metal contamination. The mean values for each study per country and sample size were used to computed the weighted mean per country. Thereafter, the weighted means were used to compute graphs of metal concentration per country.

## Results

### Distribution of studies in Africa

**Fig. 2:**
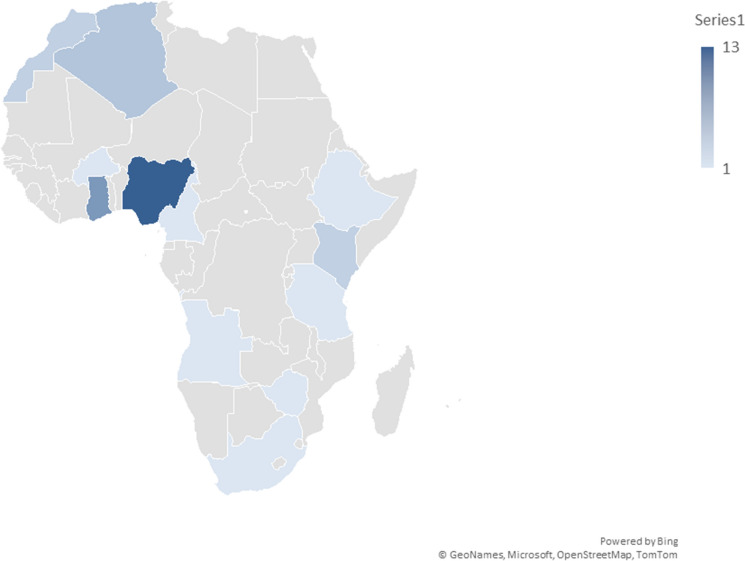
Distribution of studies across Africa on total concentration of heavy metals in urban soils

Figure 2 shows the distribution of the screened journal articles across various African countries and provides insights into the geographic focus of the research around heavy metal distribution. Nigeria stands out with 13 occurrences, indicating that more studies were conducted in this country than in others. Other countries such as Ghana (8 studies), Algeria (4 studies) and Kenya and Morocco with three studies each. Some countries had only one study, indicating minimal engagement with the subject of heavy metal contamination. However, these studies were only conducted in 12 out of the 54 countries (22%) of Africa. The lack of studies from most of the countries, including Egypt, indicates lack of research focus on heavy metal contamination of urban soils, in Africa.

### Frequency analysis

**Fig. 3:**
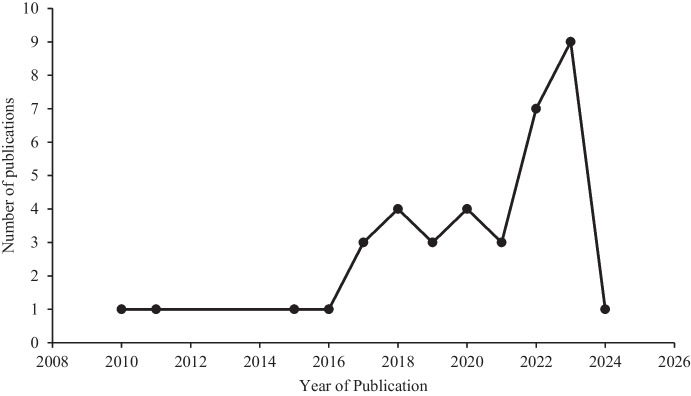
Trend of publications on heavy metals concentration in urban soils

Figure 3 depicts the trend in the number of publications over the years. The number of publications remained consistently low between 2010 and 2016, with one publication each year, while a slight increase was observed in 2017, with three publications, followed by a significant increase in 2018 (4 publications). The number of publications increased from three in 2019 to a peak of four publications in 2020. This pattern was followed by a decrease in 2021 with three publications, followed by a significant increase in 2022 with seven publications, and peaked in 2023 with nine publications. In 2024, there was a sharp decrease to one publication.

### Overview of studies identified

Table 3 summarises the total concentrations of toxic heavy metals reported across 38 studies conducted in urban soils of Africa between 2010 and 2024. The table includes data from North, West, East, Central, and Southern Africa, covering a range of anthropogenic sources, extraction and analytical techniques, and study sample sizes. Across the reviewed studies, the reported metals include arsenic (As), cadmium (Cd), chromium (Cr), nickel (Ni), lead (Pb), and mercury (Hg). Total metal concentrations varied widely among countries and study locations. The concentrations of As ranged from < 0.005 to 54.3 mg kg^−1^, while Cd ranged from 0.00056 to 54.6 mg kg^−1^. The Cr concentrations ranged between 0.023 to 660 mg kg^−1^, while Ni concentrations ranged from 0.002 to 564 mg kg^−1^. Lead concentrations were highly variable, ranging from 0.033 to 3012 mg kg^−1^, while Hg was less frequently quantified, with concentrations reported between 0.045 and 19.3 mg kg^−1^ in the few studies that measured it. A range of extraction and analytical methods was used across studies. Microwave digestion was the most frequently used extraction method and was used in combination with inductively coupled plasm spectroscopy (ICP-MS, ICP-OES, ICP-AES) or atomic absorption spectroscopy (AAS) for quantification of heavy metal concentrations. The X-ray fluorescence (XRF) was also used for direct soil analysis in only a few studies, while only one study used a hot plate digestion method. The reported sources of heavy metals varied by study and included vehicle emissions, industrial activities, domestic and municipal waste, agricultural inputs, e-waste recycling, mining, wastewater irrigation, combustion sources, and natural geological contributions. Sample sizes (n) ranged from 3 to 131 individual soil samples depending on the study.

### Method of heavy metal extraction

**Fig. 4:**
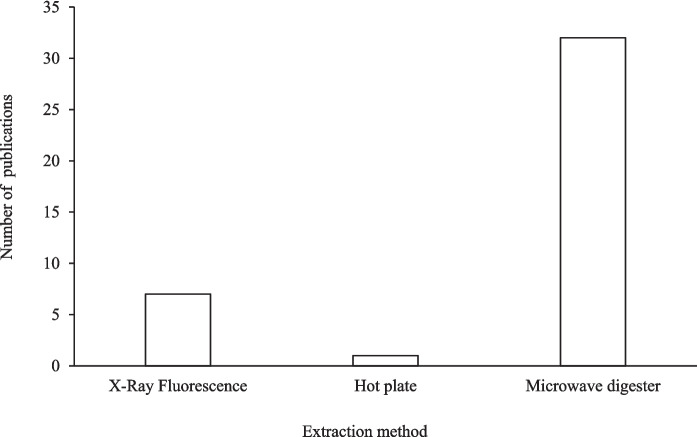
Number of publications per extraction methods for heavy metals concentration in the reviewed studies

Figure 4 depicts the methods used for extracting the heavy metals from urban soils of Africa. The most used method involves microwave digestion, with 32 occurrences in the database, emphasising the reliance placed on this method by researchers. The hot plate was only used once, with the X-ray fluorescence appearing in 7 studies.

### Adoption trends of different analytical methods

**Fig. 5:**
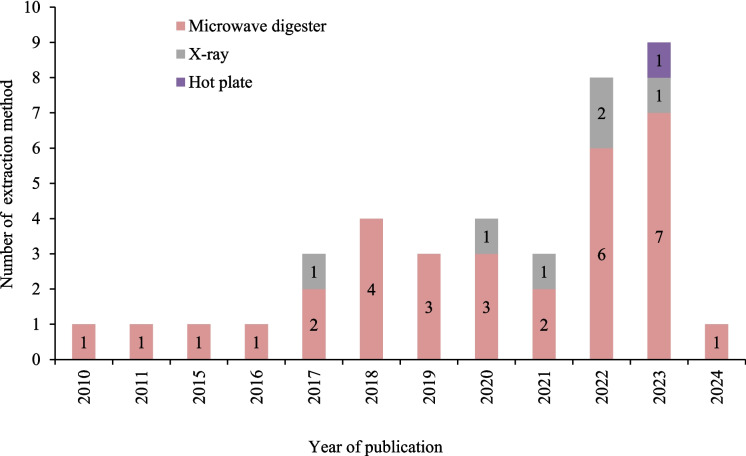
Number of extraction methods used for determination of heavy metals (2010–2024)

Figure 5 shows a trend in the gradual adoption of emerging extraction methods for heavy metal extraction from urban soils (2010 to 2024), as reflected in the publication studies. It is evident that microwave digestion has been the dominant method throughout the study period. In 2017, the X-ray fluorescence method was introduced and only one study used hot plate digestion for heavy metal extraction in 2023. However, the microwave digestion method remained the prevailing technique throughout the period covered by this study.

Identified source of heavy metals in urban soils.

**Fig. 6:**
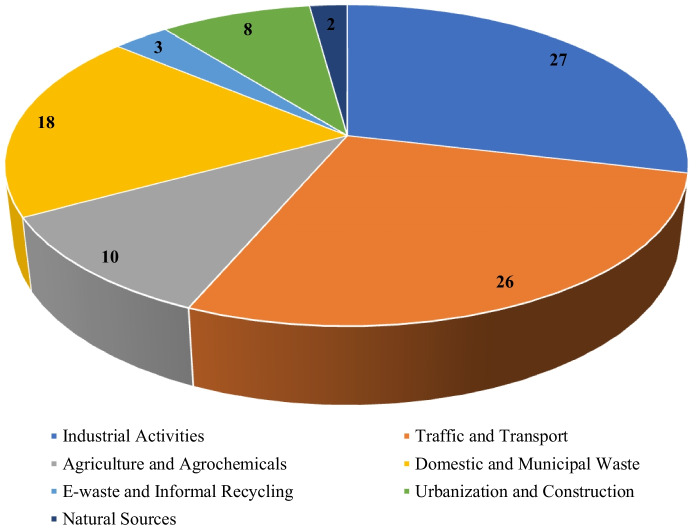
Proportion of the sources of heavy metals in the reviewed studies

The distribution of sources of heavy metals is presented in Fig. 6. Industrial activities were the largest contributors, accounting for 27%, while traffic and transport contributed 26%. The combination of the two represented over half of the observed sources of pollution. Domestic and municipal waste contributed 18%, while agriculture and agrochemicals accounted for 10%. Lesser contributions were observed from urbanisation and construction (8%), e-waste and informal recycling (3%), and natural sources (2%).

### Population per country

**Fig. 7:**
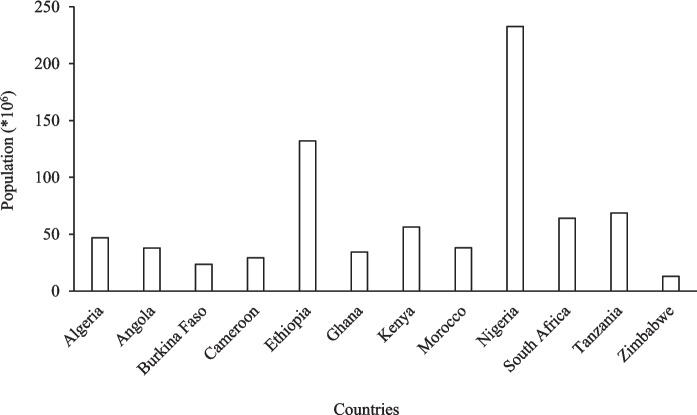
Global population of African countries (StatSA)

The results show clear variation in population among the selected African countries (Fig. 7). Nigeria recorded the highest population (232 million), followed by Ethiopia (132 million), while Zimbabwe had the lowest (13 million). South Africa and Tanzania were moderately high (64—68 million), with Kenya, Algeria, Angola, Ghana, and Morocco ranging between 34 to 56 million.

### Total heavy metals concentration in different countries

The highest concentration of As was recorded in Ghana (29 mg kg^−1^), followed by South Africa (16.0 mg kg^−1^) and Burkina Faso (12.0 mg kg^−1^). Lower concentrations were observed in Morroco (7.26 mg kg^−1^), Kenya (4.86 mg kg^−1^) and Algeria. Other countries, including Nigeria, Ethiopia, Tanzania, and Cameroon, Angola, did not report As concentration in urban soils. Cadmium (Cd) concentrations were generally low across countries, with the exception of Ghana (25.0 mg kg^−1^). Lead concentration was highest in Burkina Faso with a weighted mean of 3012 mg kg^−1^. Other countries like Cameroon (146 mg kg^−1^), Zimbabwe (99.4 mg kg^−1^), Nigeria (93.6 mg kg^−1^), and Ghana (88.48 mg kg^−1^) also reported higher concentrations of Pb, than the threshold. Chromium (Cr) was widely reported across the countries, and were all lower than the threshold, with Ghana showing the highest weighted mean (98.5 mg kg^−1^), followed by Zimbabwe (82.0 mg kg^−1^), Ethiopia (77.3 mg kg^−1^), and Burkina Faso (65.5 mg kg^−1^). Morocco and Kenya reported low Cr concentrations (27.3 and 22.9 mg kg^−1^). Whereas Tanzania, South Africa, Cameroon, and Angola did not report Cr concentration in urban soils. Nickel (Ni) concentrations were highest in Ghana (256 mg kg^−1^), followed by Ethiopia (62.7 mg kg^−1^) and Cameroon (35.2 mg kg^−1^), which were the only ones that were higher than the threshold. South Africa and Zimbabwe did not report Ni concentrations, and values from Angola were extremely low (< 0.05 mg kg^−1^). Mercury data were limited, with only a few countries reporting Hg concentrations. Kenya reported the highest weighted mean Hg concentration (14.1 mg kg^−1^), which was the only one with higher concentration than the threshold, while Ghana and Nigeria reported lower concentrations of Hg (0.09 and 0.01 mg kg^−1^, respectively). Morocco, Algeria, South Africa, Zimbabwe, Ethiopia, Tanzania, Cameroon, Burkina Faso, and Angola did not report Hg measurements.

## Discussion

### Geographic distribution in research

**Fig. 8:**
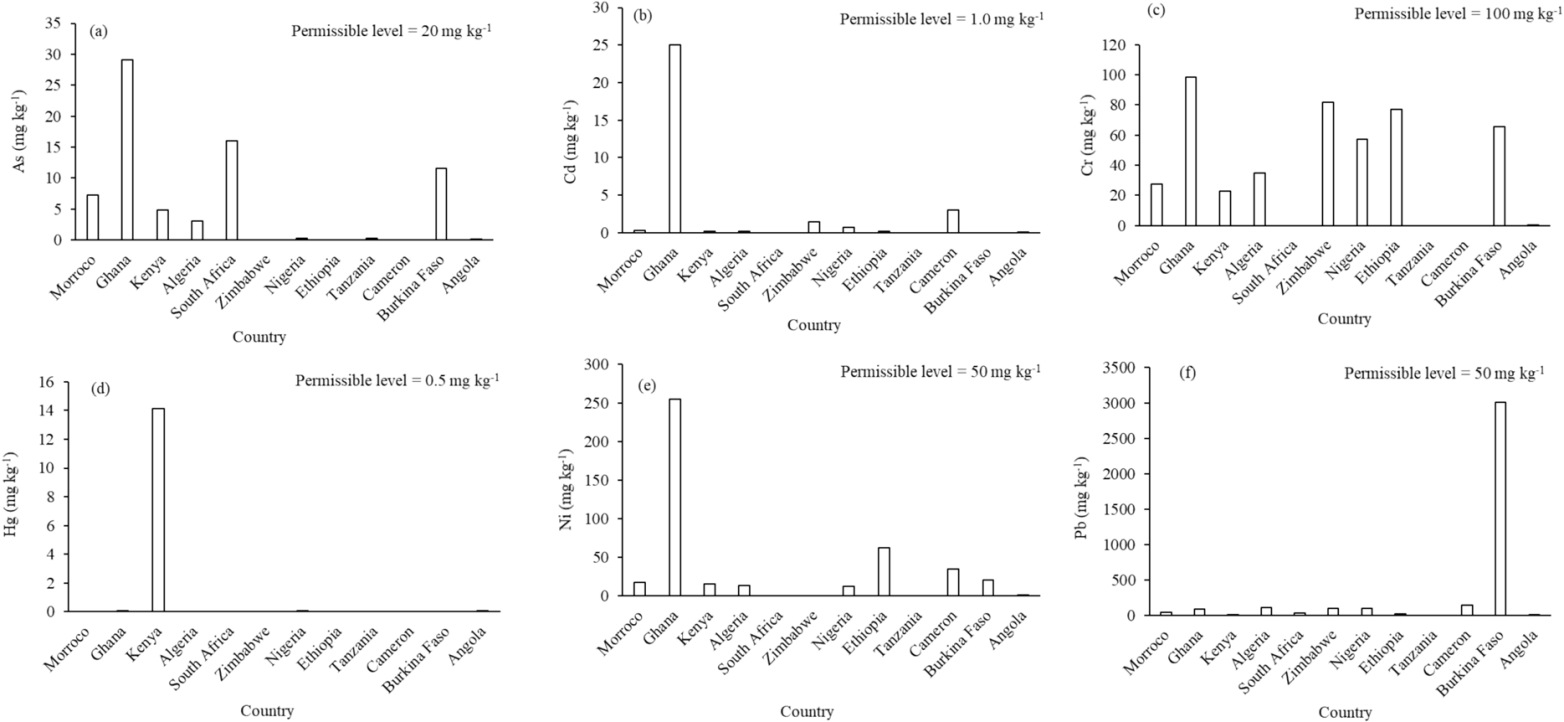
Weighted mean of total heavy metal concentration (mg kg^−1^) across different countries in Africa. The permissible levels are based on FAO (2004) permissible levels in soils

Findings from this systematic review demonstrate that heavy metal contamination in urban soils is widespread across Africa, although evidence remains fragmented. West Africa, particularly Nigeria and Ghana, had the highest reports on heavy metal concentrations in urban soils, reflecting a stronger research focus on metal contaminations in these countries. This spatial imbalance in reporting also reflected differences in urbanisation rate and industrial activity, with countries such as Nigeria and Ghana experiencing faster urban growth and higher informal industrial density (Agbabiaka et al., 2025; Agyabeng et al., 2022), which increases the potential of contamination. However, large parts of the continent (78% of the countries), including Egypt, Sudan, Uganda, Rwanda, Botswana and Mozambique, showed no published studies on heavy metals in urban soils. Given the rapid urbanisation taking place across Africa (Oduro et al., 2015), the scarcity of data in many countries raises concerns about undocumented contamination hotspots and limitations in the continent's environmental monitoring capacity. While the weighted mean concentrations of several heavy metals, may not be equally or above thresholds (Fig. 8), the elevated contamination indicated that these metals need to be monitored in urban soils across African countries. Lead (Pb) demonstrated a wide range and highest concentration, with Burkina Faso recording extremely high levels (3012 mg kg^−1^), largely linked to surrounding activities such as mining and improper waste disposal (Sako et al., 2023). However, this finding could be limited and biased due to the sample size of the country. The elevated Pb concentrations observed in Ghana (88.5 mg kg^−1^), Nigeria (93.5 mg kg^−1^), Zimbabwe (99.4 mg kg^−1^) and Cameroon (146 mg kg^−1^), which were above the permissible (50 mg kg^−1^) (FAO, 2004), were consistent with contributions from industrial emissions, informal automobile workshops, and historical use of Pb containing materials. These countries are also among the mostly urbanised ones in Africa, where high population density increases human exposure pathways through soil, dust, and food chain transfer. When compared to the thresholds, the higher weighted concentration of As, Cr, Cd Pb and Ni in Ghana, Pb in Burkina Faso and Hg in Kenya, indicates that the accumulation of these metals could be country-specific, and that Ghana currently has the greatest challenge from the documented studies. Cadmium (Cd) similarly showed pronounced variability, with Ghana (25.1 mg kg^−1^) showing the highest concentrations, aligning with global analyses indicating Cd as a major contaminant in soils (Hou et al., 2025). Chromium (Cr) and nickel (Ni) were reported more consistently across countries and below the permissible level (100 mg kg⁻^1^) (FAO, 2004), with Ghana (98.5 mg kg^−1^), Zimbabwe (82.0 mg kg^−1^), and Ethiopia (77.3 mg kg^−1^) showing the highest weighted mean concentrations than other countries. These patterns reflected the cumulative influence of metal-intensive industries, informal mechanical operations, and waste disposal practices common in highly populated urban areas (Ferreira et al., 2016; Li et al., 2022; Mehr et al., 2017). Consequently, countries with faster urban expansion rate and extensive informal industrial sectors appear more prone to elevated contamination and higher exposure risks. While the toxic metals studied here were either not reported or lower than the thresholds in most countries, including South Africa, this could be as a result of only one study on urban soils in this study, and this suggests that more studies may need to be carried out to unearth potential toxic metal hot-spots, considering the extent of rural–urban migration, industrialisation, waste management challenges, in informal settlements (Majumder & Rahman, 2023). It is also important to note that the findings were based on weighted means, which could include individual concentrations that could be equal to or higher than the thresholds.

### Sources of contaminations

The observed fluctuations in heavy metal concentrations among countries highlight the significant impact of anthropogenic activities. Industrial activities and vehicular emissions collectively constituted over 50% of the identified sources, consistent with global urban pollution patterns (Adimalla, 2020). Vehicle-induced contamination, fossil fuel burning, urban development, informal industrial activities, and electronic waste recycling collectively worsen the metal accumulation in urban soils. Despite e-waste recycling constituting merely 3% of the identified studies, it emerged as a significant localised source of severe contamination, especially in Ghana, where elevated levels of Pb (88.5 mg kg^−1^), Cd (25.1 mg kg^−1^), Ni (256 mg kg^−1^), and As (29.0 mg kg^−1^) were documented in soils. The disposal of domestic and municipal solid waste (18%) highlights the necessity of efficient waste management systems, considering that inadequately disposed solid waste significantly contributes to urban soil contamination (Frazer-Williams & Sankoh, 2024; Kulkarni & Anantharama, 2024). Variations in soil properties, land utilisation, and deposition mechanisms further account for the differences reported across countries. Several studies conducted in informal urban settlements, automotive clusters, and industrial zones where recurrent and unregulated emissions generate localised hotspots (Table 3). Peri-urban agricultural soils in Kenya and Ethiopia exhibited pollution from wastewater irrigation, sludge application, and industrial drainage (Addis et al., 2025; Ahogle et al., 2023), prompting concerns over potential crop uptake and direct exposure to farming households. These findings align with previous data indicating that peri-urban agriculture in Africa frequently functions in proximity to pollution sources.

Informal industrial activities particularly automotive workshops, e-waste recycling, and artisanal metal processing are repeatedly associated with the highest concentrations (Nduka et al., 2015; Ogbeide & Henry, 2024; Talabi et al., 2023). Their proximity to residential areas and urban agricultural lands intensifies exposure risks for vulnerable groups. Industrial operations such as metal processing, manufacturing, petrochemical refining, and waste incineration are often situated near residential zones, increasing the likelihood of continuous deposition of Pb, Cr, Cd, and Ni into surrounding soils. Traffic emissions accounted for 26% of the sources of metal inputs, including Pb, Zn, Cd, and Ni, through tyre wear, brake abrasion, exhaust fumes, and lubricating oil leaks. The dominance of industrial and traffic-related sources corresponds with patterns observed in other rapidly urbanising regions (Adimalla, 2020; Pan et al., 2018). Domestic and municipal waste (18%) reflects ongoing challenges in waste management across African cities, including open dumping, unregulated landfills, and informal waste burning. Agricultural and agrochemical sources (10%) indicate contamination arising from fertilisers, pesticides, wastewater irrigation, and livestock waste in peri-urban zones. Urbanisation and construction activities (8%) also contribute to contamination through demolition waste, building materials, and legacy pollutants. Whereas natural geological sources accounted for only 2%, confirming that heavy metal contamination in African urban soils is overwhelmingly anthropogenic. This evidence highlights an urgent need for coordinated monitoring systems and targeted remediation interventions across African urban areas. Establishing long-term monitoring frameworks would improve detection of contamination trends and support policy development, particularly in countries lacking baseline soil data. In addition, strengthening urban planning, enforcing waste-handling regulations, and improving management of informal industrial activities are critical steps towards reducing contamination. Overall, heavy metal pollution in urban soils of Africa remains an understudied environmental challenge with significant implications for public health, food safety, and urban ecosystem resilience.

### Methodological trends and limitations

Methodological difference may also partly explain differences in reported heavy metals concentrations. Although microwave digestion was the predominant extraction method and is widely recognised for its precision, some studies used XRF or hot-plate digestion, which differ in extraction efficiency and detection limits (Caporale et al., 2018). The absence of consistent protocols across studies limits direct comparability of concentrations and may influence risk estimations. This fragmentation was similarly noted in systematic evaluations regarding heavy metal contamination in Asia and Europe (Pan et al., 2018; Tong et al., 2020). Furthermore, inconsistent reporting of mean values, concentration ranges, and sample sizes could potentially limit the standardising of findings across studies. The growing utilisation of X-ray fluorescence (XRF) indicates a shift towards swift and economical screening methods that could be more appropriate for extensive monitoring. Notwithstanding methodological advancements, the absence of standardisation in sampling design and extraction techniques across studies hinders cross-country comparisons.

## Conclusion

The findings from this review demonstrate that heavy metal contamination in African urban soils is persistent, primarily resulting from anthropogenic activities, and poses a significant threat to the environment. Nonetheless, the evidence is fragmented and unevenly distributed, with no documented studied in the majority of African countries, limit the ability for accurate conclusions across the African continent. Despite these limitations, clear patterns emerge regarding localised hotspots, dominant contamination sources, and the metals of highest concern. Lead (Pb), Cr, Ni, and Cd consistently appear as major contaminants, often exceedingly internationally recognised permissible thresholds, which suggests widespread environmental and human health risks. Industrial activities and traffic emissions accounted for more than half of the identified contamination sources, while domestic waste disposal, e-waste recycling, and agricultural inputs also contributed substantially. The findings indicate that urban areas in Africa, particularly children and urban farmers, could encounter higher exposure risks owing to the close proximity of residential, agricultural, and industrial land uses. There is a need to establish and enforce standardised heavy metals monitoring frameworks and promote specific remediation strategies to protect soil quality and safeguard vulnerable populations in urban soils of Africa. This review identifies significant research gaps, noting the insufficient assessment and quantification of heavy metals in urban soils of Africa, which position urban soils of Africa as highly vulnerable zones.

## Data Availability

Data will be made available from the corresponding author upon reasonable request.
